# Accuracy of Physical Assessment in Nursing for Cervical Spine Joint Pain and Stiffness: Pilot Study Protocol

**DOI:** 10.2196/31878

**Published:** 2021-12-17

**Authors:** Bruno Soares, Raquel Fonseca, Patrícia Fonseca, Paulo Alves

**Affiliations:** 1 Instituto Ciências da Saúde Universidade Católica Portuguesa Porto Portugal; 2 Faculdade Medicina Dentária Universidade Católica Portuguesa Viseu Portugal

**Keywords:** nursing process, nursing assessment, pain, referred pain, range of motion, neck pain, stomatognathic system, viscerosomatic reflexes, cervical spine dysfunction

## Abstract

**Background:**

Cervical spine dysfunction is a condition with high personal, social, and economic impact worldwide. Although its etiology is described as multifactorial, there is a need for further clarification. The literature has demonstrated the anatomical, physiological, and pathophysiological relationship among the cervical spine, temporomandibular joint, and visceral system. To guide and contribute to the accuracy of the physical assessment performed by nurses, we will study the influence of the stomatognathic system and viscerosomatic reflexes on pain and joint stiffness of the cervical spine.

**Objective:**

The aim of this study is to describe a pilot study protocol to investigate the influence of the stomatognathic system and viscerosomatic reflexes on cervical structures.

**Methods:**

A pilot study with a quasi-experimental design was conducted with 50 volunteers from the university population of the Universidade Católica Portuguesa-Porto. We studied the influence of changes in the usual intercuspation, the occlusal deprogramming, and the pressure stimulus of the reflex skin region of the ilium/colon in the cervical spine. This study was divided into 2 phases. In the first phase, we performed the kinematic and pain analysis during the passive mobilization of the upper cervical spine using the Motion Capture System at the Motion Capture Laboratory at UCP-Porto and the Visual Analog Scale. In the second phase, we evaluated the pain threshold on palpation of the erector neck muscles and the structures of the stomatognathic system using algometry. The influence of viscerosomatic reflexes on the structures of the stomatognathic system was also analyzed.

**Results:**

Selection and preparation of the data collection site, acquisition of materials, constitution of the sample group and data collection were completed. The analysis of the results is being carried out.

**Conclusions:**

The data from this study will allow for the detection of the possible influence of the stomatognathic system and viscerosomatic reflexes on pain and range of motion of the upper cervical spine, providing data for future randomized studies. We have also identified potential limitations of this study.

**International Registered Report Identifier (IRRID):**

RR1-10.2196/31878

## Introduction

Cervical spine dysfunction (CSD) is a pathological condition of the spine with high prevalence worldwide that is expected to increase in the future. It dramatically impacts individuals, families, and society. This condition is characterized by pain phenomena, functional disability, decreased quality of life, social activity and mental health impacts, and increased mortality; CSD therefore has both individual and societal costs [1-4].

According to estimates from the Global Burden of Disease Study 2017 [1], both the prevalence and burden of CSD are high worldwide, with a global prevalence of 288.7 million cases, an incidence of 65.3 million cases, and 25.6 million years lived with disability.

Although pain and joint stiffness are associated with CSD, imaging studies in a population with this condition have not identified any specific lesion, leaving the etiology of this condition unknown, resulting in inaccurate diagnoses [5-8]. This may be the reason why therapeutic interventions tend to have insufficient results [1,2,9].

The cervical dysfunction etiology is thus described as multifactorial, mediated by central neuronal commands resulting from complex biological interactions of the local or distant structures of the cervical segment; this creates great variability in the course and clinical severity of the condition [1,3-5,10].

It is therefore important to integrate these components when evaluating the person with CSD to identify the mechanisms underlying their health condition and apply interventions adjusted to their physical condition. In this way, nurses should use strategies consistent with scientific methodology when conducting physical assessments of CSD patients, providing reliability to their approach and aiding their decision-making [11,12].

The assessment of the person with CSD is assumed to be objective, using a set of measurement methodologies consisting of physical assessment, specific pain analysis, and complementary means of diagnosis [5,13,14].

From the multifactorial perspective of CSD, the function and dysfunctions of the stomatognathic system, due to reciprocal synergistic action, alter the neck’s correct functioning [15-18].

This coactivation coordination between the stomatognathic system and the cervical spine appears to be related to the neuronal network centers that regulate the muscles of these body segments and are mediated by the cervical motor sensory system and the trigeminal nerve [17,19].

On the other hand, nociceptive hyperexcitability can promote the development or maintenance of chronic pain, such as by triggering painful reflex disorders [20].

Other neurological phenomena that seem to influence this relationship are the viscerosomatic reflexes. These are the result of harmful afferent signals of visceral origin that converge in somatic structures by common innervation or by induction of neuronal plasticity of the central, peripheral, and autonomic nervous system, involving multiple organs and body structures [21-25]. Regarding the interdependence of the cervical spine and the stomatognathic system, visceral sensory convergence through the vagus nerve at the trigeminal and spinothalamic tract of the C1-C2 level has an important role in the functioning of the upper cervical segments through the integration of the converging entrances of somatic and visceral organs [26].

Given these connections between different body systems, this study aims to analyze the influence of the stomatognathic system and viscerosomatic reflexes on pain and joint stiffness of the UCS. Two research questions were defined in this study: (1) What is the influence of the stomatognathic system and viscerosomatic reflexes on pain and joint stiffness in UCS? (2) What is the influence of viscerosomatic reflexes on the stomatognathic system?

The main objective of this study is to contribute to the clarification of the pain and joint stiffness etiology of the cervical spine to increase the accuracy of physical assessment performed by nurses.

To achieve the aforementioned major objective, the following objectives were defined: (1) to identify the influence of the stomatognathic system on pain and cervical mobility, (2) to identify the influence of viscerosomatic reflexes on pain and cervical mobility, and (3) to identify the influence of viscerosomatic reflexes on the stomatognathic system.

## Methods

To our best knowledge and based on the literature review conducted, no prior studies investigated the same variables with similar methodologies. Therefore, this study presents a preclinical/pilot study profile.

In this investigation, we adopted the methodology of a quasi-experimental study with an interrupted time series design.

### Data Collection Phases

The study will consist of 2 data collection phases:

- Phase I: Kinematic analysis. Assessment of the rotational range of motion of the UCS and pain during passive mobilization
- Phase II: Palpation of neck and oral muscles. Assessment of pain threshold on palpation (algometry)

### The Population Under Study and Constitution of the Sample

The study population consisted of university students at the Academic Federation of Porto as this population is made up of adults of different age groups who tend to be healthy and exhibit similar behaviors, habits, and lifestyles. From this population, a nonprobabilistic sample by voluntary response was drawn, composed of 50 volunteers. The volunteers included students, professors, and nonacademic staff.

The study was publicized by placing posters on the Universidade Católica Portuguesa-Porto (UCP-Porto) premises, and a call for volunteers was made on the UCP-Porto Facebook page. Volunteers registered by sending an email indicating their name, including their contact details, and declaring their interest in participating in the study.

Afterwards, researchers contacted participants to confirm their interest in participating, apply the inclusion and exclusion criteria, and schedule the data collection if they were accepted for the sample group, guaranteeing ethical principles and confidentiality.

The criteria for inclusion in the sample were:

1. Being 18 years of age or older.
2. Agreeing to participate in the study.

The exclusion criteria for the formulation of the study sample were:

1. Receiving pharmacological therapy (analgesics, anti-inflammatory drugs, and/or muscle relaxants).
2. The existence of neuromuscular pathology, congenital alteration, pathological condition in the acute phase, or functional disturbances of the cervical spine and/or mandibular that make the application of variables or passive mobilization of the cervical spine unfeasible.
3. A history of bone fractures; surgery to the cervical spine, skull, and/or mandibular; or cancer.
4. Undergoing a physical rehabilitation program.

### Operationalization of Variables

The variables will be operationalized as follows:

- Range of motion of the UCS: variable operationalized using the Motion Capture System, which allows for the measurement of the range of motion from 0° to 90°.
- Pain during mobilization of the UCS: variable operationalized through an open-ended question corresponding to a numerical value between 0 and 10, as recommended by the Visual Analog Scale (VAS).
- Pain threshold on palpation of the erector neck muscles: variable operationalized in two dimensions, pain and pressure force. Pain will be operationalized through an open-ended question, corresponding to a numerical value between 0 and 10, as recommended by the VAS. The pressure force will be operationalized through algometry corresponding to a numerical value between 0 and 4315 kPa.

### Interventions

#### Occlusal Deprogramming

**Figure 1:**
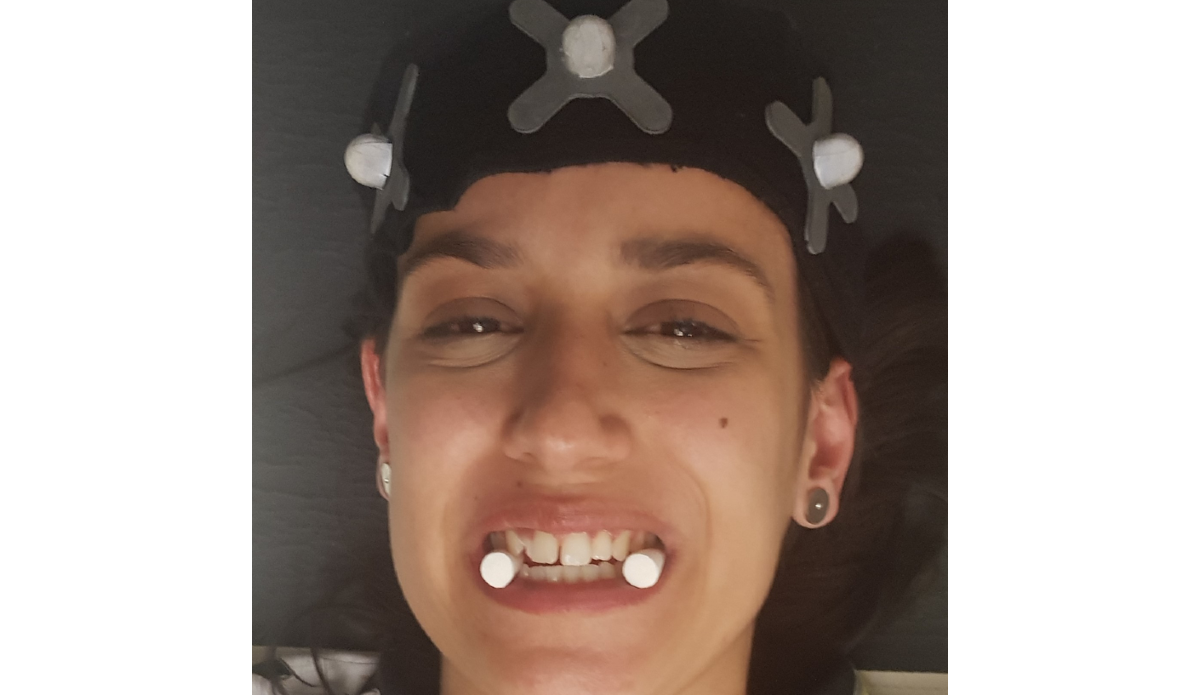
Neuromuscular occlusal deprogramming. The moment of compression of the cotton balls by the participant.

Neuromuscular occlusal deprogramming aims to reduce the action of masticatory muscles on the mandible, promoting its centric position within the temporomandibular joint. We chose to use cotton balls for this study in a simple and economical methodology with an immediate neuromuscular result, allowing the necessary time for the evaluations to be performed [27]. This intervention strategy consists of placing a pair of cotton balls bilaterally at the height of the premolars and asking the participant to vigorously compress them for approximately 3 to 5 minutes, as shown in Figure 1.

**Figure 2:**
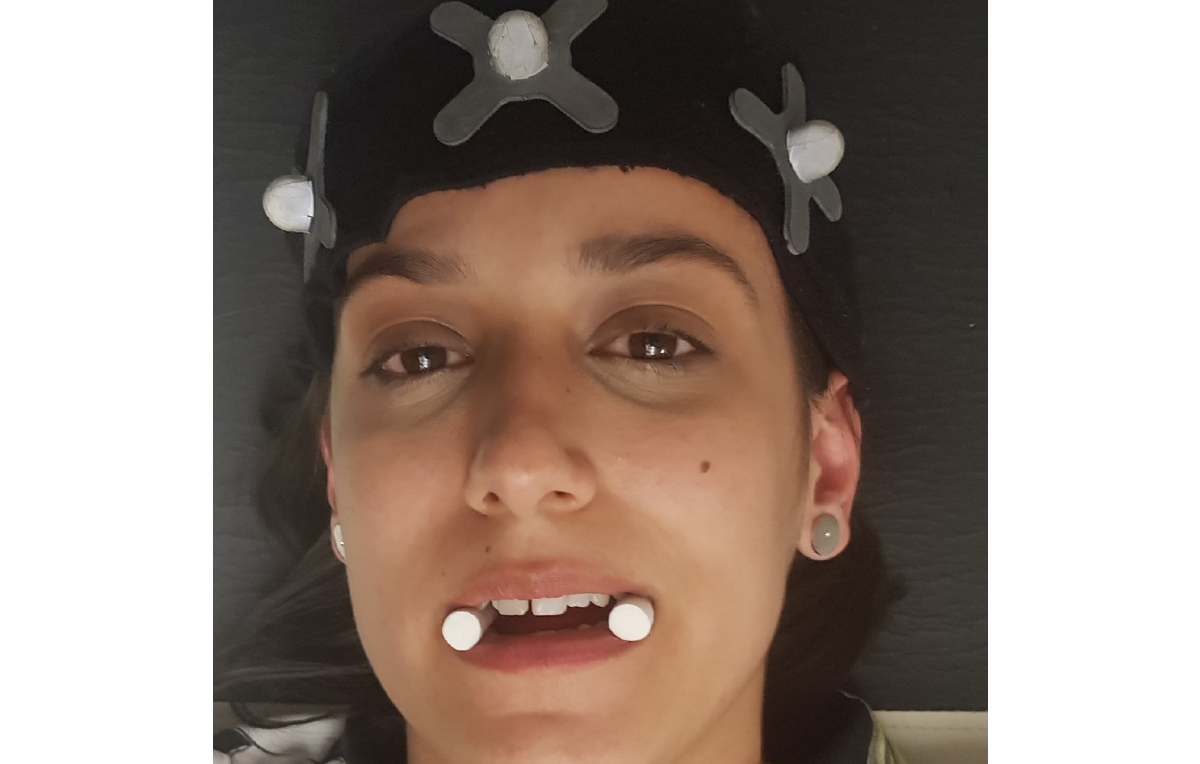
The change in the usual intercuspation with placement of cotton balls bilaterally at the level of the premolars.

To understand if the change in the usual intercuspation altered the pain and joint stiffness of the UCS, before promoting the occlusal deprogramming, when placing the cotton rolls at the premolar level, a kinematic evaluation of the UCS was conducted (Figure 2).

#### Pressure Stimulus of the Reflex Cutaneous Region of the Ilium/Colon

**Figure 3:**
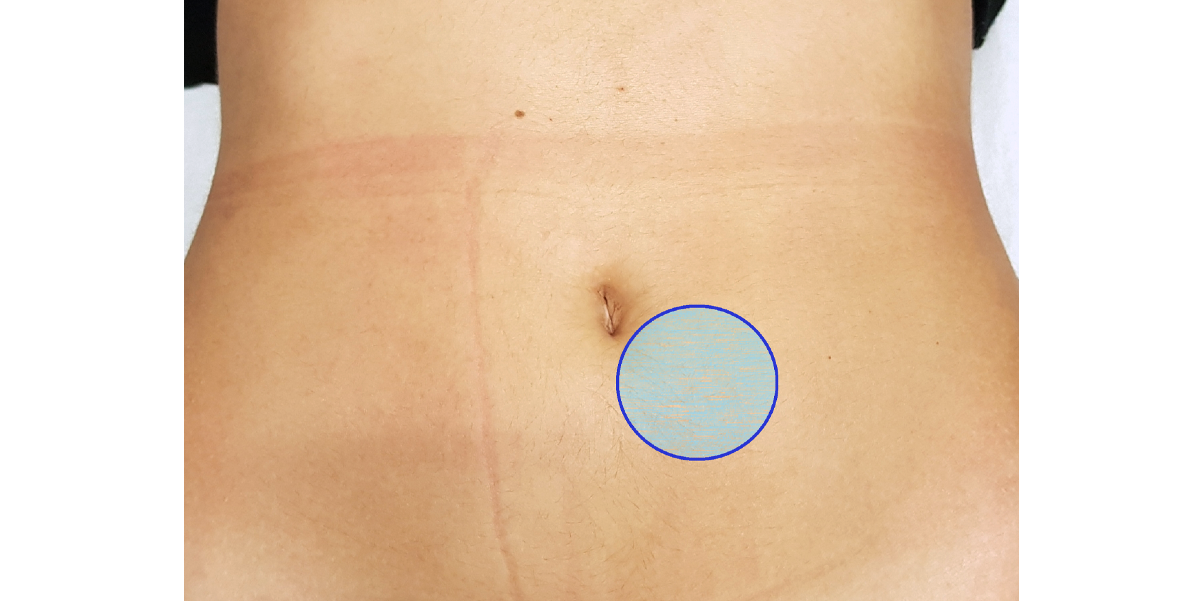
Reflex cutaneous region of the ilium/colon.

No physical assessment methodologies capable of promoting the assessment of the viscerosomatic reflexes influence on musculoskeletal structures were found in a literature review. In this sense, a pressure stimulus was performed on the abdominal cutaneous region described by Arendt-Nielsen et al [28], corresponding to the ilium/colon reflex, as depicted in Figure 3.

**Figure 4:**
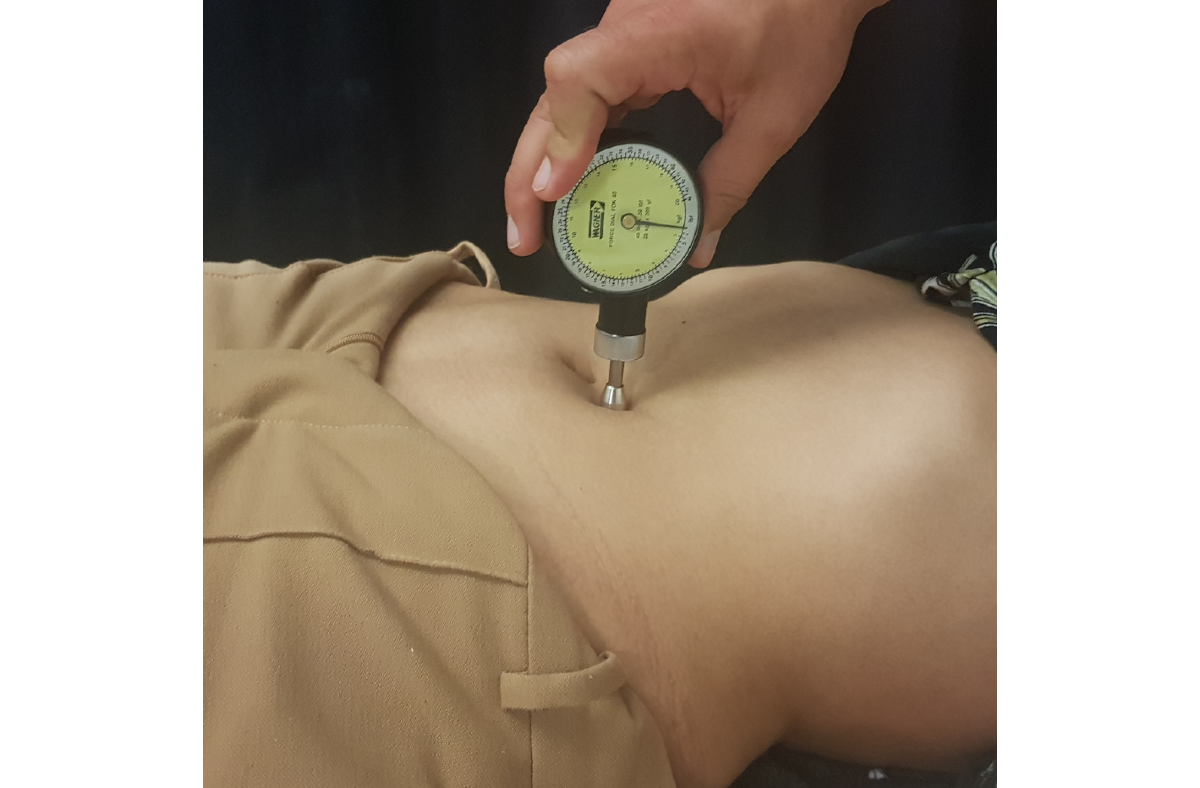
Tactile stimulation of the reflex skin region of the ilium/colon.

The application of a pressure of 196 kPa in this anatomical region was determined to stimulate the superficial tissues using an algometer (Force Dial FDK/FDN 40, Wagner Instruments). We did this to ensure accuracy and standardization of the stimulus in Test 3 (Figure 4).

### Data Collection Instruments

#### Motion Capture System

**Figure 5:**
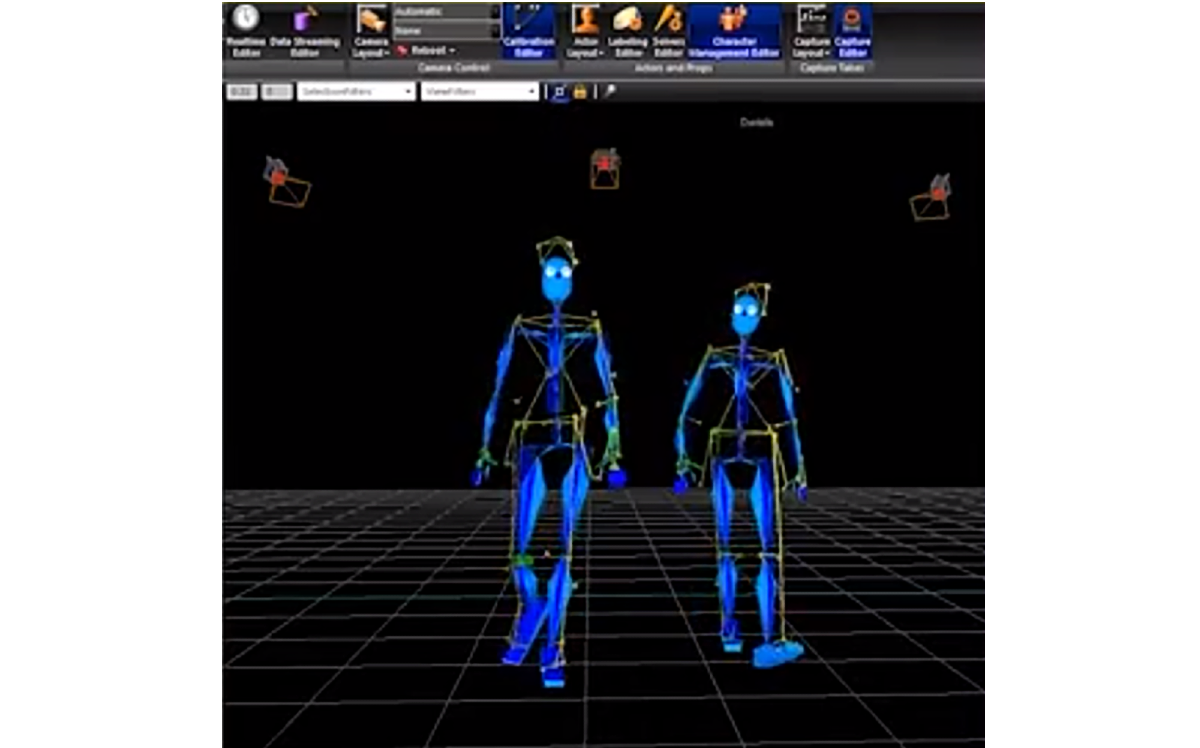
A 3D image reproduced from the Motion Capture System sensor data collection.

**Figure 6:**
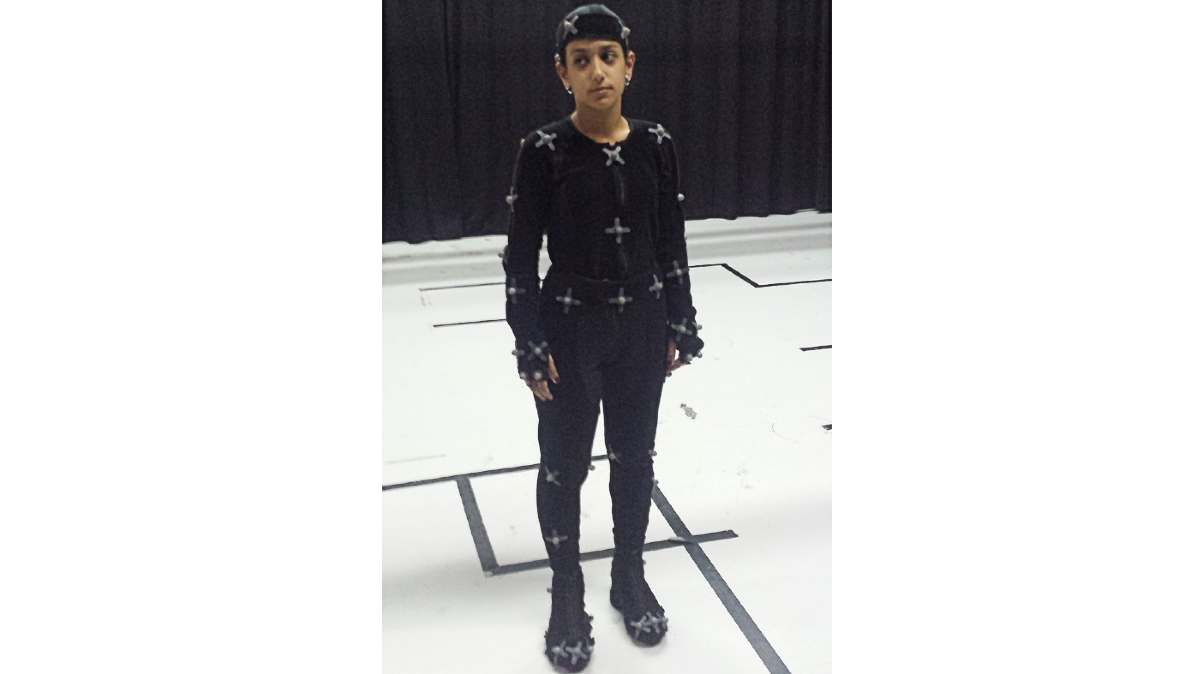
Fabric suits worn by participants on which Motion Capture System sensors were placed.

The Motion Capture System at the Motion Capture Laboratory at UCP-Porto allows for the capture of 3D motion and has been used for the kinematic analysis of the human body in clinical evaluations and in the study of biomechanics (Figure 5). Data collection is performed in a computerized room with data collection cameras around the room. The cameras are connected to a computer in a control system that allows for the visualization of the collected data and its registration. The collected data come from sensors that are placed on the body of the study participants. Participants must wear a fabric suit that allows for different sensor allocations and the standardization of their placement between participants (Figure 6). The data provided by this assessment methodology are enhanced as they are in 3D, while data collected by goniometry are in 2D; this methodology also does not require the intervention of the researcher to collect the data, allowing them greater freedom to promote interventions [29,30].

This assessment methodology allows for less evaluator interference but maintains the same reliability as goniometry [30,31], the gold standard in range of motion assessment [13,32].

**Figure 7:**
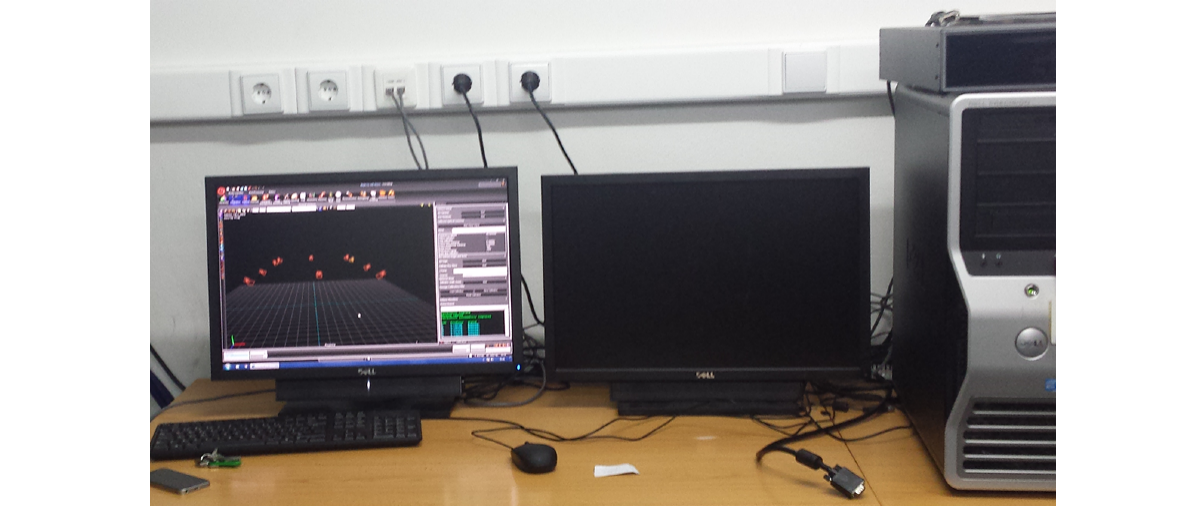
Motion Capture Central Processing Unit (CPU) Room at UCP-Porto.

**Figure 8:**
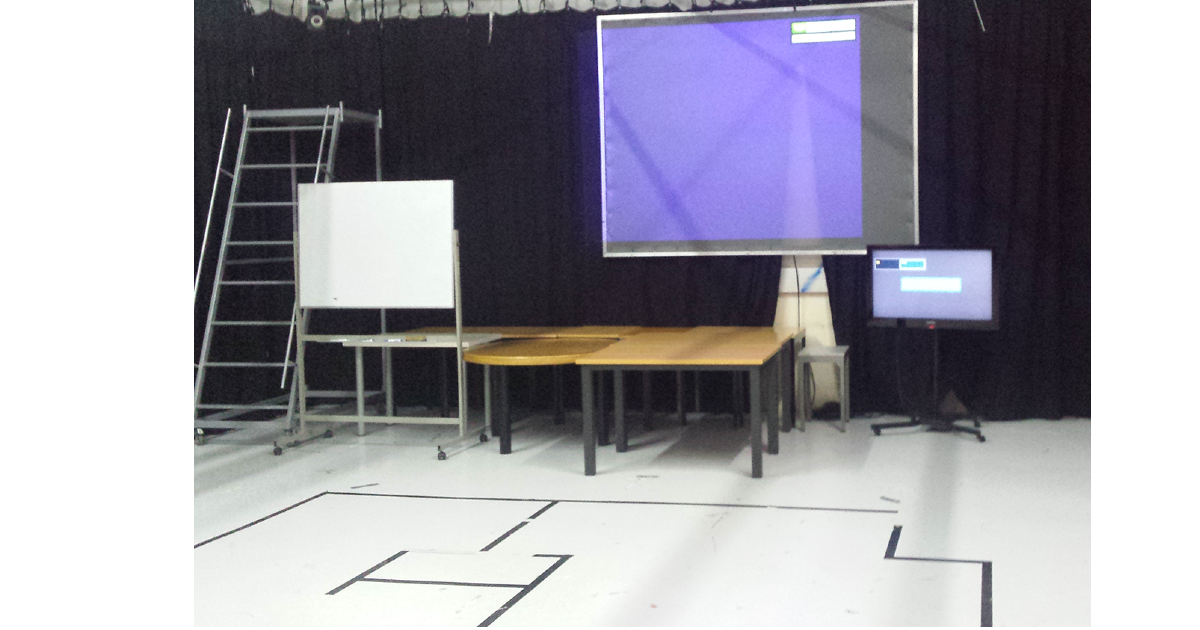
UCP-Porto Motion Capture Room.

As this is a 3D system, it allows for the collection of movement in the x-axis, y-axis, and z-axis. For this study, only data from the z-axis were counted because the analysis took place on the longitudinal axis of the cervical spine. Data collection was conducted at the Motion Capture Laboratory at UCP-Porto, a laboratory financed by the Foundation for Science and Technology, where the Motion Capture System is located (Figures 7 and 8).

#### Algometer

**Figure 9:**
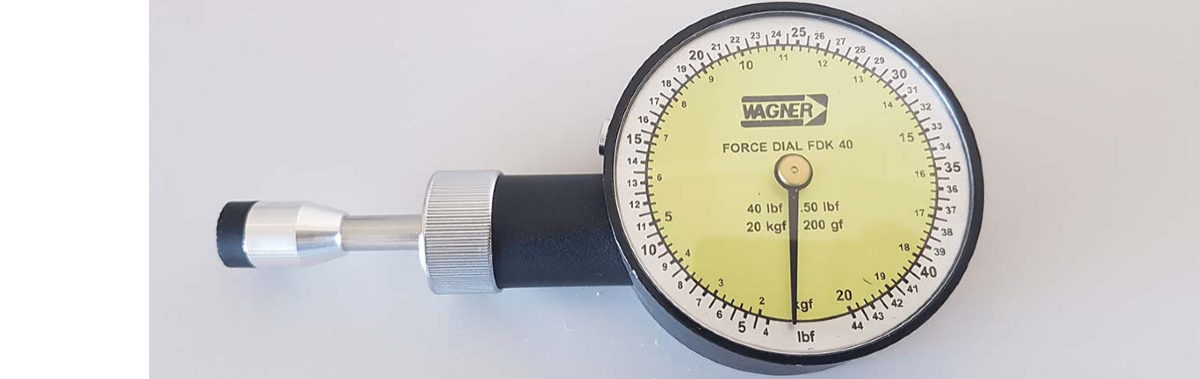
The algometer used in the study (Force Dial FDK/FDN 40, Wagner Instruments).

The palpation of body structures is one of the methodologies used in the physical assessment, allowing for the examination and perception of the condition and characteristics of the evaluated structures, the existence of hypersensitivity or hyposensitivity, the presence or absence of injury, as well as the detailed evaluation of each body structure. In applying this methodology, one should start with the minimum pressure and increase the intensity of its application according to the characteristics of the structures and the tolerance of the participant or patient [13,14]. Algometry allows for the measurement of the force produced and is considered a reliable methodology [33]. In this study, the Force Dial FDK/FDN 40 algometer was used, allowing for the measurement of the applied force in kg/cm^2^ (Figure 9).

#### Visual Analog Scale (VAS)

**Figure 10:**
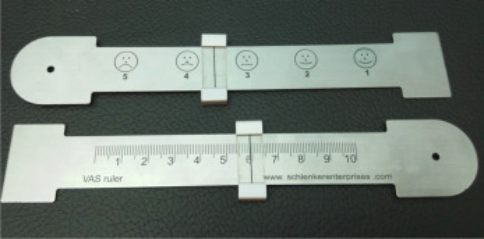
Visual Analog Scale for pain measurement [34].

For the self-assessment of the pain intensity experienced at different phases by the participants, we used the VAS, shown in Figure 10 [34].

### Performance and Data Collection Protocols

To ensure maximum reliability in data collection, we ensured that the researcher had more than 10 years of experience in manual therapy and assessment of spinal mobility, following literature guidelines [35,36]. To gain familiarity with the data collection methodology, handling of materials, and standardization of the assessment, the researcher performed pretests on more than 30 volunteers.

Data were collected between June and July 2019. There was a gap of 1 week between phase I and phase II.

#### Environmental Conditions

Data collection took place in two separate rooms. In both rooms, the environmental conditions were stabilized using (1) artificial lighting, allowing for the stabilization of light intensity, and (2) a heater, allowing for the stabilization of the room temperature between 20 °C and 22 °C.

#### Phase I Procedures: Kinematic Analysis

In phase I, kinematic and pain evaluation during mobilization of the first (C1) and second (C2) cervical vertebrae was performed. We used the Motion Capture System to measure the range of motion.

Upon arrival of the participant at the Motion Capture Laboratory, we proceeded to collect or confirm the following data:

- Name
- Eligibility for participation based on the inclusion and exclusion criteria
- Informed consent

We then:

- Explained all study procedures.
- Presented data collection materials.
- Answered remaining questions.
- Signed and delivered the informed consent forms to the participant and the researcher.
- Completed the sociodemographic characterization survey. If the participant met the conditions for participation and accepted of their own free will, the procedures for the operationalization of the study would start.

**Figure 11:**
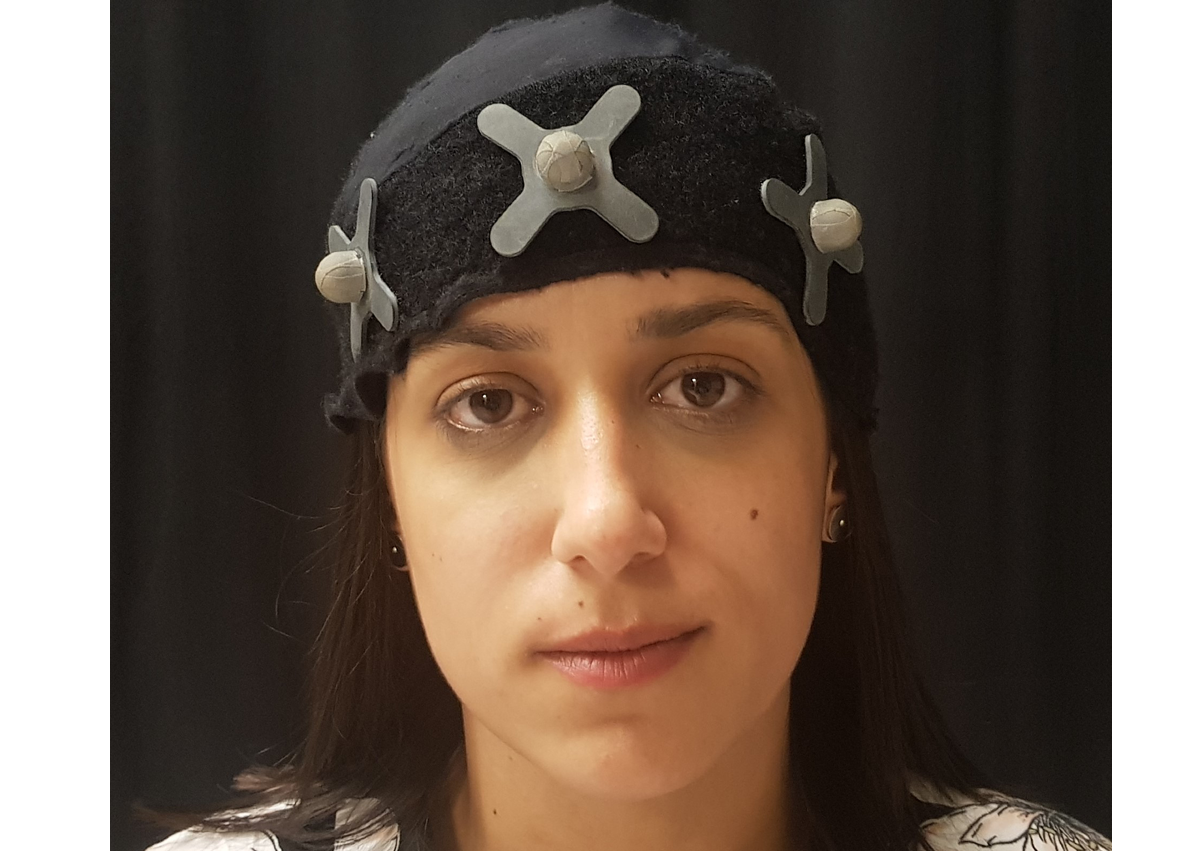
Fabric helmet with sensors for data collection.

**Figure 12:**
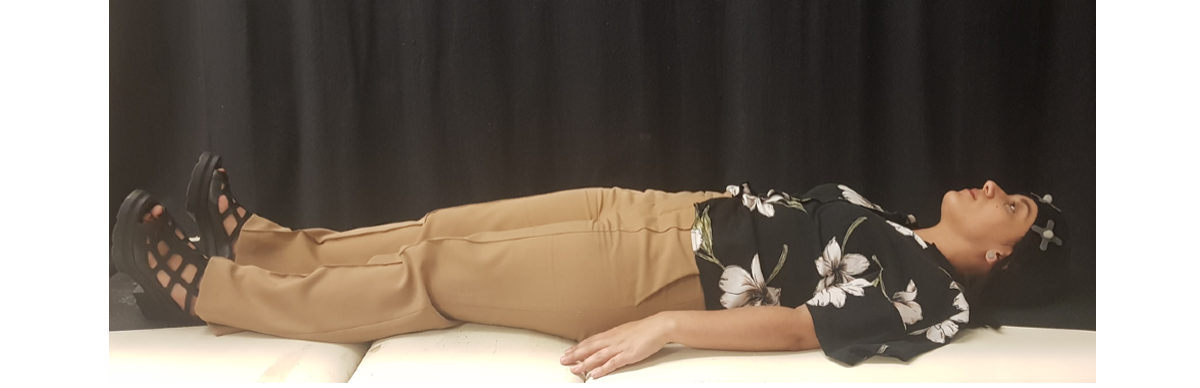
Data collection position, allowing for maximum relaxation of the cervical spine structures.

During the preparation for data collection, a cloth helmet with three sensors was applied to each participant’s head (Figure 11). Subsequently, the participant was placed in the supine position on a gurney as this position allows for maximum relaxation of the cervical spine structures (Figure 12).

**Figure 13:**
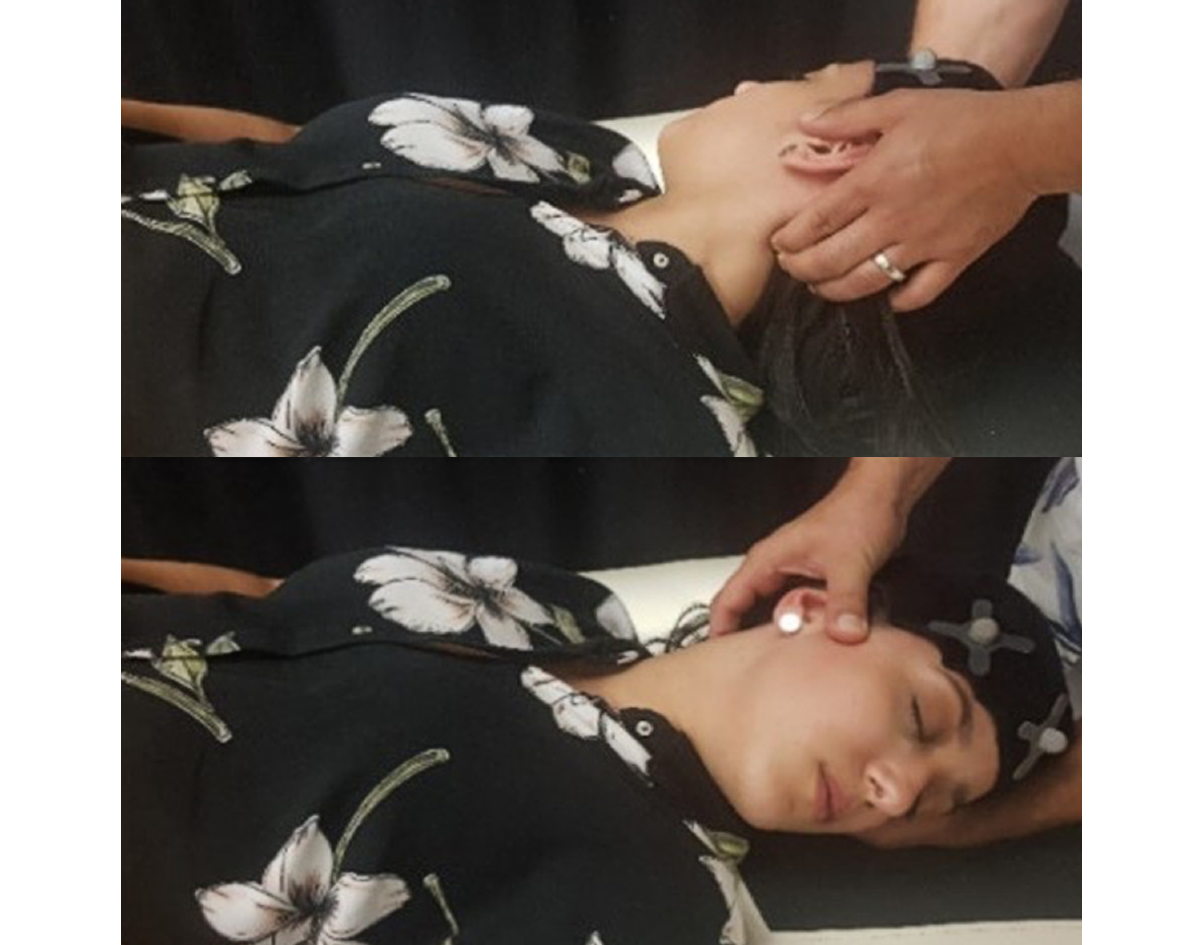
Passive mobilization of the upper cervical spine (UCS), applying a rotational movement to the right and to the left.

Data collection involving C1 and C2 passive joint mobilization (Figure 13) occurred in five phases: (1) Initial Assessment, (2) Test 1, (3) Test 2, (4) Initial Assessment 2, and (5) Test 3 (Table 1). Between Test 2 and Initial Assessment 2, an interval of 15 to 20 minutes took place, to promote washout, with the objective of having the participant in their usual condition to assess the influence of Test 3.

**Table 1:** Description of the 5 phases in which data collection occurred during C1 and C2 passive joint mobilization.

Phase		Description
**Initial Assessment**		Assessment during maximum body relaxation, with the teeth in intercuspation without load
	Test 1	Change of usual intercuspation with cotton balls
	Test 2	Occlusal deprogramming
**Initial Assessment 2**		Assessment during maximum body relaxation, with the teeth in intercuspation without load
	Test 3	Application of a pressure stimulus to the reflex cutaneous region of the ilium/colon

#### Phase I Protocol

The steps of the phase I protocol are as follows:

1. Initial Assessment: Passive mobilization of the cervical spine was performed to assess the rotational range of motion and pain perceived at the time of assessment, at C1 and C2 levels, with the teeth in intercuspation without load (antagonistic teeth touching without a load exerting force). A total of 3 measurements were taken and were later averaged. Between each measurement was a pause of 20 seconds.
2. Test 1: The researcher performed cervical passive mobilization to assess the rotational range of motion and pain, at C1 and C2 levels, promoting the alteration of the usual intercuspation with placement of cotton balls between the dental arches. A total of 3 measurements were taken and were later averaged. Between each measurement was a pause of 20 seconds.
3. Test 2: The researcher performed cervical passive mobilization to assess the rotational range of motion and pain, at C1 and C2 levels, after performing the procedures for occlusal deprogramming. These procedures include placement of cotton balls between the dental arches, vigorous compression of cotton balls for 3 to 5 minutes, removal of cotton balls, and placement of the patient in a position of maximum relaxation with intercuspation without load. A total of 3 measurements were taken and were later averaged. Between each measurement was a pause of 20 seconds.
4. Pause: a pause of 15 to 20 minutes was taken to promote washout.
5. Initial Reassessment 2: The researcher performed cervical passive mobilization to assess the rotational range of motion and pain perceived at the time of assessment, at C1 and C2 levels, with intercuspation without load (antagonistic teeth touching without exerting force). A total of 3 measurements were taken and were later averaged. Between each measurement was a pause of 20 seconds.
6. Test 3: The researcher performed cervical passive mobilization to assess the rotational range of motion and pain, at C1 and C2 levels, with the application of a pressure stimulus of less than 196 kPa in the reflex cutaneous region of the ilium/colon continuously throughout the evaluation. A total of 3 measurements were taken and were later averaged. Between each measurement was a pause of 20 seconds.

This evaluation phase lasted 30 minutes per participant.

#### Phase II Procedures: Palpation of Neck and Orofacial Muscles

Data collection took place at least 1 week after phase I to rule out any type of influence from previously applied interventions.

Upon arriving, the participant was reminded of the procedures to be performed and the algometer and the VAS were presented again, enabling participants to characterize any type of pain they might experience during data collection. After confirming their willingness to continue the study, each participant was placed in the supine position on a gurney.

The tests and procedures used were the same as in phase I, with the exception of Test 1. The exclusion of this test is because the presence of an object that prevents habitual occlusion can stimulate the muscle contraction of the stomatognathic system and UCS, altering their “normal” condition and consequently altering their painful sensitivity to palpation.

**Figure 14:**
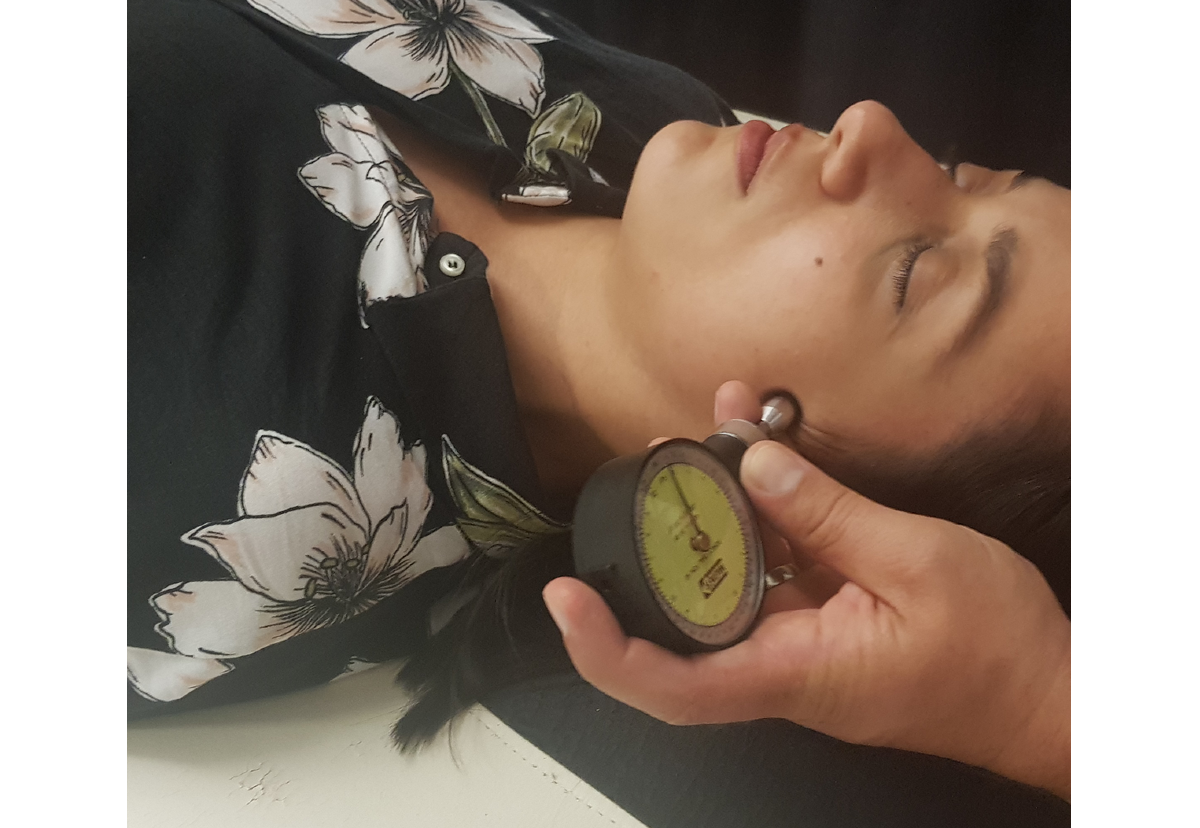
Pain threshold assessment on palpation.

The following erector muscles of the neck and stomatognathic system were evaluated in phase II (Figure 14):

- Trapezius
- Suboccipital musculature
- Sternocleidomastoid
- Temporal (anterior, middle, and posterior portions)
- Masseter (Upper to origin, body, and insertion)
- Ear-jaw articulation
- Medial pterygoid site

**Figure 15:**
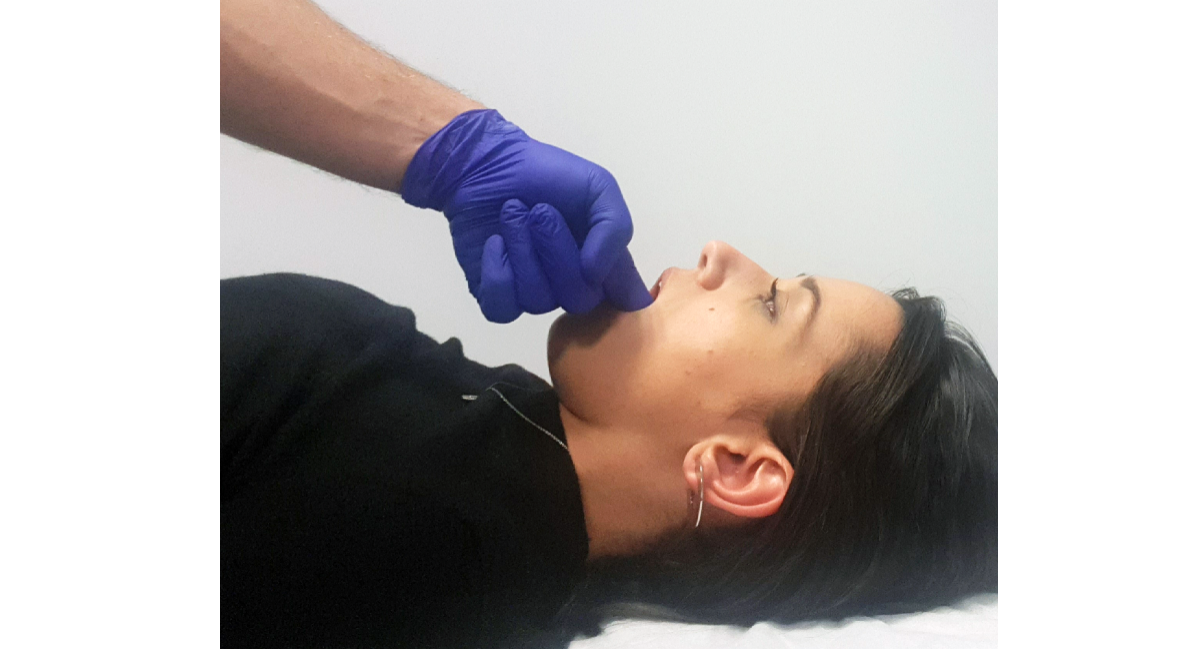
Palpation of the medial pterygoid site.

Due to its anatomical location, it is not possible to use the algometer to assess the medial pterygoid site; therefore, it was only evaluated by direct palpation with the finger (Figure 15).

#### Phase II Protocol

The steps of the phase II protocol are as follows:

1. Initial Assessment: The researcher performed palpation of the erector neck muscles and of the stomatognathic system structures with intercuspation without load, using the AVS to characterize the resulting pain. Algometry was used to measure the pressure applied to the evaluated muscles A total of 3 measurements were taken and were later averaged. Between each measurement was a pause of 20 seconds.
2. Test 2: The researcher performed palpation of the erector neck muscles and of the stomatognathic system structures after occlusal deprogramming, using the VAS to characterize the resulting pain. Algometry was used to measure the pressure applied to the evaluated muscles. A total of 3 measurements were taken and were later averaged. Between each measurement was a pause of 20 seconds.
3. Pause: A pause of 15 to 20 minutes was taken to promote washout.
4. Initial Reassessment 2: The researcher performed palpation of the erector neck muscles and of stomatognathic system structures with intercuspation with load, using the AVS to characterize the resulting pain. Algometry was used to measure the pressure performed on the evaluated muscles. A total of 3 measurements were taken and were later averaged. Between each measurement was a pause of 20 seconds.
5. Test 3: The researcher performed palpation of the erector neck muscles and of the stomatognathic system structures using the VAS to characterize the resulting pain during tactile compression of the reflex region of the ilium/colon (pressure less than 196 kPa). This was controlled with algometry. A total of 3 measurements were taken and were later averaged. Between each measurement was a pause of 20 seconds.

This evaluation phase lasted 45 minutes per participant.

### Ethical Procedure

This study was reviewed and approved by the Ethics Committee of the Regional Center of Porto, from the Catholic University of Portugal (CE.219.[11].2018).

To guarantee the safety of the participants and the confidentiality of the data and information from the study, an informed consent form containing the purpose of the study and interventions to which participants would be subjected was delivered for reading and signing. The signed document was delivered to the researcher.

### Data Procedures

After collecting data from our sample, they will be entered into Excel (Microsoft Corporation) and then transferred to R (R Foundation for Statistical Computing), a free software for statistical analysis and graph construction, which is considered a variant of the S language. This program was developed by the R Foundation for Statistical Computing, with the aim of creating a tool for free use.

Descriptive statistics will be used to analyze the data relating to the characterization of the sample. This includes analysis of frequency distributions (for qualitative and discrete quantitative variables) and descriptive measures (minimum, maximum, mean, median, quartiles, standard deviation, coefficient of variation and Fisher asymmetry coefficient for discrete or continuous quantitative variables). This data will also be presented in graphical format using histograms and boxplots for better visualization of the results.

For the inferential statistics of the variables (range of motion, pain associated with passive mobilization, pressure exerted in the assessment of the pain threshold, and pain experienced by the pressure stimulus), the following procedures will be used:

- To perform result comparisons at the time of evaluation, it will be necessary to first check that the data have a normal distribution using the Shapiro-Wilk normality test (*P*<.001).
- The results will be compared using the Friedman test (*P*<.001), also known as analysis of variance in Friedman orders, because the data come from related samples (the same participants in the various evaluation phases).
- Due to the completion of the Friedman test, it will be necessary to proceed with multiple comparisons. As the samples are paired (since they are the same participants in both evaluation phases), the Wilcoxon test (*P*<.001) will be used, allowing for the identification of the differences between the evaluation phases.
- To analyze the relationship between the variables at different phases of evaluation, we will use the Spearman order correlation coefficient (*P*<.001).

## Results

The selection and preparation of the data collection site, the acquisition of materials, the constitution of the sample group, and data collection have been completed. The results are being analyzed.

## Discussion

The data from this study will allow for the observation of the possible influence of the stomatognathic system and viscerosomatic reflexes on pain and range of motion of the UCS, providing data for future randomized studies.

### Limitations

As this is a pilot study, the objective is not to generalize the results, but to describe the behavior of the variables and contribute data for the development of future randomized studies.

No clinical diagnoses were made regarding the cervical spine, stomatognathic, or visceral system condition, allowing for the stratification of the participants.

Tactile stimulation of the reflex cutaneous region of the ilium/colon was a methodology designed for the study because the local physiology of this skin region was correlated with viscerosomatic reflexes. The physiological phenomena of this stimulus must be studied to better understand its mechanism of action.

## Abbreviations

CSD: cervical spine dysfunction
UCP-Porto: Universidade Católica Portuguesa-Porto
UCS: upper cervical spine
VAS: Visual Analog Scale
